# P-591. Infection with Campylobacter and Risk of Guillain-Barré Syndrome: a Meta-Analysis

**DOI:** 10.1093/ofid/ofaf695.805

**Published:** 2026-01-11

**Authors:** Rima Shrestha, Hunter Pool, Moni Roy, Hannah Welter, Daniel Heydari, Sharjeel Ahmad

**Affiliations:** University of Illinois College of Medicine at Peoria, Peoria, IL; UIC-University of Illinois College of Medicine Peoria (UICOMP), Peoria, Illinois; UIC-University of Illinois College of Medicine Peoria (UICOMP), Peoria, Illinois; UIC-University of Illinois College of Medicine Peoria (UICOMP), Peoria, Illinois; UIC-University of Illinois College of Medicine Peoria (UICOMP), Peoria, Illinois; UIC-University of Illinois College of Medicine Peoria (UICOMP), Peoria, Illinois

## Abstract

**Background:**

*Campylobacter* is one of the common bacterial agents for gastroenteritis worldwide. Guillain-Barré Syndrome (GBS), an autoimmune disorder of the peripheral nervous system, can be triggered by *Campylobacter* infection. Though 20-31% of GBS cases are estimated to be attributed to *Campylobacter,* the updated quantification of *Campylobacter* infections and their association with GBS is crucial for informing risk prevention strategies.

**Figure:**
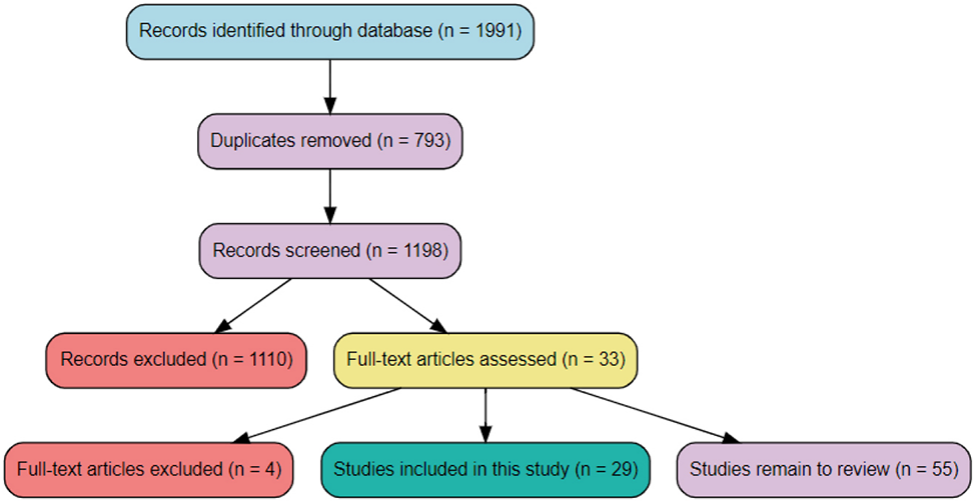
The flow diagram illustrates the study selection process for inclusion in the analysis conducted in the COVIDENCE software.

**Figure 2.**
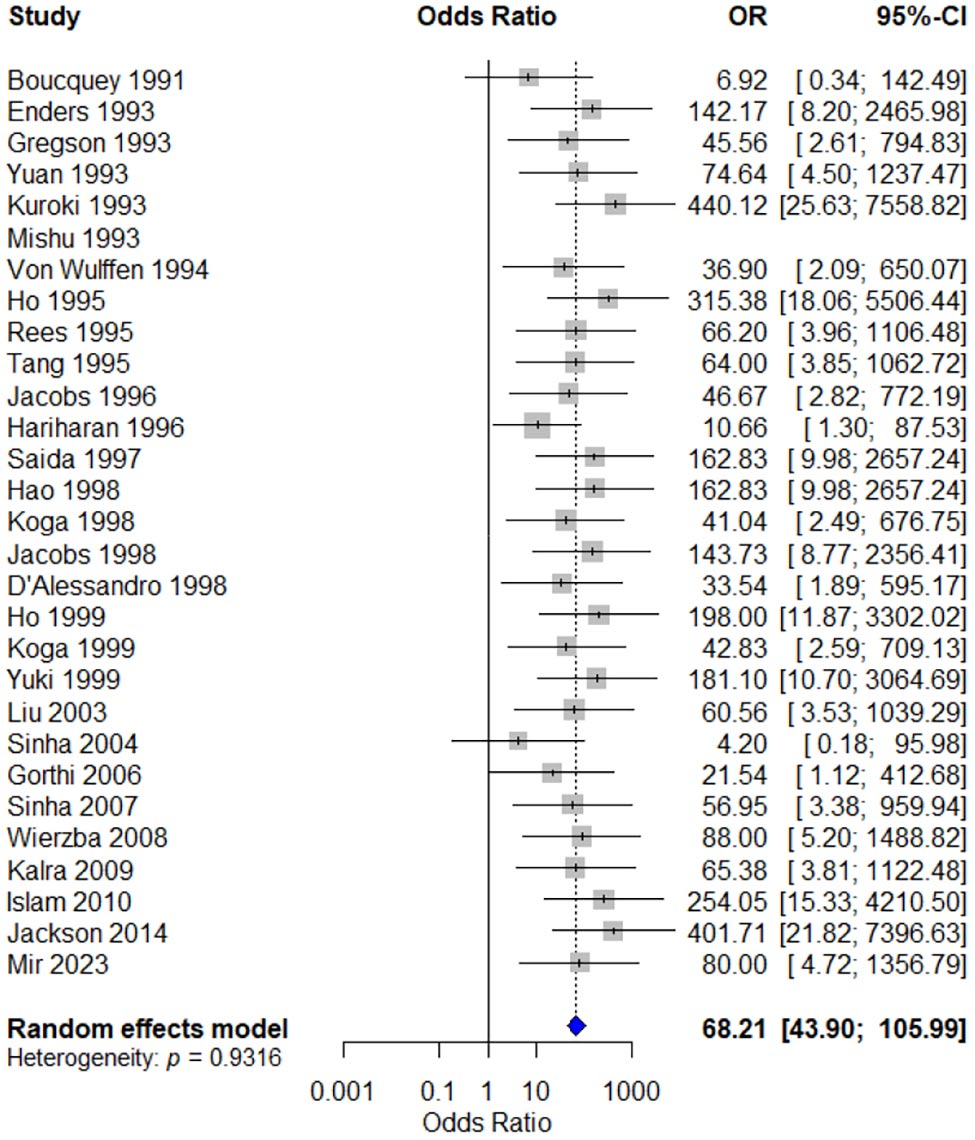
Forest plot illustrating the odds ratio of Campylobacter positivity in Guillain–Barré syndrome (GBS) cases compared to controls.

**Methods:**

A peer-reviewed paper published from 1990-2024 was obtained from various databases. MeSH terms created using inclusion: patients of any age, gender, and ethnicity with confirmed *Campylobacter* infection through stool culture, serology, or PCR, and developed GBS from *Campylobacter* infection; and exclusion: recent Influenza vaccine within 2 weeks, studies with insufficient data to calculate risk, and any other disease associated GBS (e.g. COVID-19) criteria. Manuscripts were systematically reviewed in the COVIDENCE software. Data (Figure 1) were extracted on characteristics, number of events, exposure status, participants' age, country, and study designs. Pooled odds ratios (ORs) were calculated from a random-effects model using R packages and R Studio. Heterogeneity, publication bias, and analysis sensitivity were evaluated.

**Results:**

Case-control studies (n=29) comprising 2621 GBS cases, 2688 controls, and 1046 GBS with *Campylobacter* infections were included. The pooled mean age of the participants was 34.33 years [95% CI: 22.65-46.02]. The pooled OR was 68.21[43.90-105.99] (Figure 2). No heterogeneity was observed (I^2^ = 0.0% [0.0%; 41.9%]; tau^2^ = 0 [0.00; 0.31]). The Egger’s test and funnel plot symmetry indicated the absence of publication bias (p = 0.31). A leave-one-out sensitivity analysis showed no single study substantially influenced the pooled effect size.

**Conclusion:**

Patients with prior *Campylobacter* infection have 68.21 times higher risk of GBS compared to uninfected individuals. This demonstrates a strong and statistically significant association between *Campylobacter* infection and the subsequent development of GBS. Further analysis for incidence, relative risk, and subgroup analysis (to be shared in the final presentation with additional review) will preserve the strength of association we found.

**Disclosures:**

Sharjeel Ahmad, MD, MPH, FACP, FIDSA, Karius, Inc: Advisor/Consultant

